# Inter-rater reliability and validity of risk of bias instrument for non-randomized studies of exposures: a study protocol

**DOI:** 10.1186/s13643-020-01291-z

**Published:** 2020-02-12

**Authors:** Maya M. Jeyaraman, Nameer Al-Yousif, Reid C. Robson, Leslie Copstein, Chakrapani Balijepalli, Kimberly Hofer, Mir S. Fazeli, Mohammed T. Ansari, Andrea C. Tricco, Rasheda Rabbani, Ahmed M. Abou-Setta

**Affiliations:** 1 The George & Fay Yee Center for Healthcare Innovation, University of Manitoba, 363-753 McDermot Avenue, Winnipeg, Manitoba R3E 0 T6 Canada; 2 Department of Community Health Sciences, University of Manitoba, Winnipeg, Canada; 3 Li Ka Shing Knowledge Institute, St. Michael’s Hospital, Unity Health Toronto, Toronto, Canada; 4 Pharmalytics Group, Vancouver, Canada; 5 Evidinno Outcomes Research Inc., Vancouver, British Columbia Canada; 6 School of Epidemiology and Public Health, Faculty of Medicine, University of Ottawa, Ottawa, Canada; 7 Epidemiology Division, Dalla Lana School of Public Health & Institute of Health, Management, and Policy Evaluation, University of Toronto, Toronto, Canada; 8 Queen’s Collaboration for Health Care Quality Joanna Briggs Institute Centre of Excellence, Queen’s University, Kingston, Ontario Canada

**Keywords:** Inter-rater reliability, Inter-consensus reliability, Concurrent validity, Risk of bias, ROB, NRS, NRSE, Cross-sectional study, Non-randomized studies

## Abstract

**Background:**

A new tool, “risk of bias (ROB) instrument for non-randomized studies of exposures (ROB-NRSE),” was recently developed. It is important to establish consistency in its application and interpretation across review teams. In addition, it is important to understand if specialized training and guidance will improve the reliability in the results of the assessments. Therefore, the objective of this cross-sectional study is to establish the inter-rater reliability (IRR), inter-consensus reliability (ICR), and concurrent validity of the new ROB-NRSE tool. Furthermore, as this is a relatively new tool, it is important to understand the barriers to using this tool (e.g., time to conduct assessments and reach consensus—evaluator burden).

**Methods:**

Reviewers from four participating centers will apprise the ROB of a sample of NRSE publications using ROB-NRSE tool in two stages. For IRR and ICR, two pairs of reviewers will assess the ROB for each NRSE publication. In the first stage, reviewers will assess the ROB without any formal guidance. In the second stage, reviewers will be provided customized training and guidance. At each stage, each pair of reviewers will resolve conflicts and arrive at a consensus. To calculate the IRR and ICR, we will use Gwet’s AC_1_ statistic.

For concurrent validity, reviewers will appraise a sample of NRSE publications using both the Newcastle-Ottawa Scale (NOS) and ROB-NRSE tool. We will analyze the concordance between the two tools for similar domains and for the overall judgments using Kendall’s tau coefficient.

To measure evaluator burden, we will assess the time taken to apply ROB-NRSE tool (without and with guidance), and the NOS. To assess the impact of customized training and guidance on the evaluator burden, we will use the generalized linear models. We will use Microsoft Excel and SAS 9.4, to manage and analyze study data, respectively.

**Discussion:**

The quality of evidence from systematic reviews that include NRSE depends partly on the study-level ROB assessments. The findings of this study will contribute to an improved understanding of ROB-NRSE and how best to use it.

## Background

Systematic reviews inform healthcare decision-making by summarizing the best available evidence [1]. Critical appraisal of the available evidence via assessment of the risk of bias (ROB) in the studies included in a systematic review plays a key role in determining the strength or quality of the systematic review [2]. Although evidence from randomized controlled trials (RCTs) is generally considered superior to that of non-randomized studies (NRS) to prove causation [2], it is important to include evidence from NRS when evidence from RCTs is indirect, imprecise, inconsistent, inapplicable, or unavailable [3, 4]. Having said that, it is important for systematic reviewers to be aware of the potential biases associated with NRS designs, and the best practices to minimize the impact of these biases on the effect estimate [5].

Many quality assessment tools are available to assess the methodological quality of non-randomized, observational studies of exposures (NRSE) [6–8] The Newcastle-Ottawa scale (NOS) is the most widely used [7] and has eight items divided into three domains (selection, comparability, and either outcome or exposure assessment for cohort and case-control studies, respectively). NOS uses a star system with a maximum of 1 star assigned to all eight items (except for one item under comparability domain, which receives a maximum of 2 stars); total scores can range between 0 to 9 stars [7]. Although the NOS is widely used, it was reported to have poor inter-rater reliability (IRR) [5].

In 2016, the Cochrane Methods Bias (CMB) group and the Cochrane Non-randomized Studies of Interventions Methods Group, along with a large team of experts, published the “Risk of Bias in Non-randomized Studies of Interventions” (ROBINS-I) [9] tool. The ROBINS-I tool guides adjudications on the risk of bias (ROB) of non-randomized studies (NRS) of interventions (NRSI), by comparing it to a hypothetical target randomized controlled trial (RCT) that the NRSI best emulates (even if the RCT would be unethical or unfeasible) [2, 9]. As ROBINS-I was developed to assess ROB in interventional studies (“intentional exposures”), there was no clarity on its usefulness in assessing ROB in NRS of environmental, nutritional, or other exposures (“unintentional exposures”) [10]. Unlike ROBINS-I, most previously available instruments neither use signaling questions nor the comparison to an ideal RCT concept [10].

In recent years, with an improved understanding of the potential effects of study design, study conduct, and study reporting, there has been a major shift from using checklists for assessing study quality (or just reporting per se), to assessing ROB [11, 12]. Study quality refers to how a study was designed to avoid systematic errors during the conduct, analysis, and interpretation/reporting of the results and conclusions. Risk of bias refers to factors that could have systematically biased the study design, interpretation, analysis, presentation, or reporting of the outcome measures. Due to the intensified focus on the field of exposure assessment by the Grading of Recommendations, Assessment, Development and Evaluation (GRADE) Working Group and the need for harmonizing the rating of NRSI and the NRSE in the context of GRADE [10], a collaborative project led by a team of experts at the University of Bristol (UK), McMaster University (Canada), and the Environmental Protection Agency (USA) proposed to create a new instrument “Risk of Bias in Non-randomized Studies of Exposures” (ROBINS-E) [10]. ROBINS-E is currently under development, and as of the end of 2019, there were no official draft versions available for review [13]. What is currently available is a preliminary tool developed by the GRADE working group named the “ROB instrument for NRS of exposures” [10, 14]; for simplicity we will use the acronym ROB-NRSE to refer to this tool. ROB-NRSE was developed by making significant semantic and conceptual modifications to the ROBINS-I tool. In 2019, a user’s guide to its application in the context of GRADE was also published [15].

The authors suggest following three steps in the evaluation of ROB using ROB instrument for NRS of exposures (ROB-NRSE) [10]:

1. Step I: Describing the systematic review question (e.g., Population, Exposure, Comparator, Outcomes—PECO)
2. Step II: Describing ideal target experiment, confounders and co-exposures
3. Step III: Outcome-specific ROB evaluation of the included studies by the raters using the ROB-NRSE [10]

Similar to ROBINS-I, ROB-NRSE is composed of seven domains to assess bias due to confounding, selection of participants, classification of exposures, departures from intended exposures, missing data, measurement of outcomes, and selection of reported results [9]. Each of the seven domains contain multiple signaling questions with five response options (*yes*, *probably yes*, *no*, *probably no*, *or no information*) to guide domain level ROB adjudications [9]. The ROB adjudications are categorized as follows: *low risk, moderate risk*, *serious risk*, *critical risk*, *or no information*.

As ROB-NRSE is the most current, publicly available version modeled after the ROBINS-I tool, we conducted this cross-sectional study to establish ample evidences on its reliability and validity in order to improve the consistency in its application and in how it is interpreted across various systematic reviews that include NRSE. Inter-rater reliability (IRR) refers to the reproducibility or consistency of decisions between two reviewers and is a necessary component of validity [16, 17]. Inter-consensus reliability (ICR) refers to the comparison of consensus assessments across pairs of reviewers in the participating centers. Concurrent validity refers to the extent to which the results of the instrument or tool can be trusted [17]. Furthermore, it is important to understand the barriers to using this tool (e.g., time to conduct assessments and reach consensus—evaluator burden).

## Methods/design

Using methods similar to those described previously for the evaluation of the ROBINS-I tool [18], an international team of experienced researchers from four participating centers will collaboratively undertake this study. The major objectives are the following:

I. Measure the IRR and ICR between reviewers when assessing ROB of NRSE using ROB-NRSE (without and with customized training and guidance)
II. Measure the concurrent validity of ROB-NRSE
III. Measure the evaluator burden (time taken to apply ROB-NRSE, time taken to arrive at a consensus, time taken to apply NOS)

In order to address the above objectives, we will conduct a cross-sectional analytical study on a sample of NRSE publications following this protocol. This protocol will be registered with the Open Science Framework (https://osf.io/). The final study manuscript will be reported according to the STROBE-cross-sectional checklist [19–21]. We plan to report any protocol amendments in the final study manuscript.

### I: Inter-rater reliability and inter-consensus reliability

Our first objective is to evaluate the IRR of ROB-NRSE at first stage, without customized training and guidance document from principal investigator, and then at the second stage, with customized training and guidance. At both stages, assessors will have access to the publicly available detailed guidance [22]. For the second stage, a customized guidance document will be developed using Microsoft word (Word v1.5, Microsoft Corp., Redmond, WA, USA), by a senior member of the team holding PhD degree (MJ). Following review and feedback by another experienced senior member of the team (MA), we will finalize the document. The guidance document will contain simplified decision rules, additional guidance for advanced concepts, and clarifications on answering signaling questions that will guide reviewers in making adjudications for each domain in ROB-NRSE tool. Once developed, we will send the guidance document to all the reviewers, for help with adjudications in the second stage of the project. Additionally, one training session (via Skype) will be organized by a trainer (MJ), who is a senior member of the team and the developer of the customized guidance document. During the training session, the trainer will review the guidance document with all the reviewers and provide clarifications. We will use the following methods to assess IRR and ICR.

#### Participating centers

We will involve two pairs of reviewers (LC, NA, RCR, CB, and KH) with varied levels of experience and academic degrees attained, from multiple research teams to assess IRR and ICR. The participating teams are as follows: (coordinating center) The Knowledge Synthesis platform, George & Fay Yee Center for Healthcare Innovation, University of Manitoba (Canada) (MJ, AMAS, LC, NA, RR); Knowledge Synthesis Team, Knowledge Translation Program, Li Ka Shing Knowledge Institute of St. Michael’s Hospital, Unity Health Toronto (Canada) (ACT and RCR); Evidinno Outcomes Research Inc. (Canada) (KH and MSF); Pharmalytics Group, Vancouver (Canada) (CB).

#### Sample size calculation

We have calculated the sample size (number of NRSE publications) required for IRR assessments (*n* = 44) by taking into account a 5% type I error, 80% statistical power, and an assumed error margin of 30% [23–25]. As suggested by Gwet [23, 24], we assumed the chance-agreement probability (*P*_e_) as zero (best-case scenario) and estimated the required sample size for IRR using the formulas and calculations available at: http://agreestat.com/blog_irr/sample_size_determination.html. We obtained the observed-agreement probability (*P*_a_) between reviewers required for sample size calculation from an initial pilot testing of 10 NRSE publications.

#### Sample selection

We propose to use a convenience sample of prospective cohort publications published in English (*n* = 44), (based on the sample size calculations) identified from a recent systematic review. We will then identify one pre-specified outcome (the primary outcome of each study), for ROB appraisals for each of the included NRSE. If a study does not report a primary outcome, the principal investigator will identify an important outcome reported in the study, for ROB appraisal. With the help of content experts, we will identify a list of confounders and important co-exposures for the specific association of interest reported in each of the included NRSE publications.

#### Data collection

After the initial pilot testing on 10 studies, we will proceed with ROB assessments for IRR. We will advise the reviewers to review the any available general guidelines for ROBINS-I provided by the developers of the ROBINS-I tool available at https://methods.cochrane.org/news/robins-i-tool. This would reflect normal practice for new researchers that have not had previous training/expertise with the tool. We will also advise all reviewers in the participating centers to read the full report of each included NRSE prior to making assessments. Reviewers will have the list of confounders and important co-exposures available during their assessments. At first, two reviewers will independently assess the ROB for the included NRSE using ROB-NRSE tool, without using any formal training or customized guidance. For each included NRSE, the two reviewers will assess the seven domains of ROB-NRSE tool as, *low ROB*, *moderate ROB*, *serious ROB*, *critical ROB*, *or no information* [9] (Table 1). At the end, the two reviewers will resolve conflicts and arrive at a consensus.

**Table 1:** ROB-NRSE tool

	Domains	Response options	Support for judgement	Review author’s decision
**1**	**Bias due to confounding**			
1.1	Is there potential for confounding of the effect of exposure in this study?			
	**If N/PN to 1**.**1**: the study can be considered to be at low risk of bias due to confounding and no further signaling questions need be considered. **If Y/PY to 1**.**1**, answer 1.2 and 1.3 to determine whether there is a need to assess time-varying confounding:			
1.2	Was the analysis based on splitting follow up time according to exposure received?			
	**If N or PN to 1**.**2**, answer questions 1.4 to 1.6, which relate to baseline confounding **If Y or PY to 1**.**2**, go to question 1.3			
1.3	Were exposure discontinuations or switches likely to be related to factors that are prognostic for the outcome?			
	**If N or PN to 1**.**3**, answer questions 1.4 to 1.6, which relate to baseline confounding **If Y/PY**, answer questions relating to both baseline and time-varying confounding (1.7 and 1.8)			
1.4	Did the authors use an appropriate analysis method that adjusted for all the important confounding areas?			
1.5	**If Y/PY to 1**.**4**: Were confounding areas that were adjusted for measured validly and reliably by the variables available in this study?			
1.6	Did the authors avoid adjusting for post-exposure variables?			
1.7	Did the authors use an appropriate analysis method that adjusted for all the critically important confounding areas and for time-varying confounding?			
1.8	**If Y or PY to 1**.**7**: Were confounding areas that were adjusted for measured validly and reliably by the variables available in this study?			
	**Risk of bias judgement**			
	Optional: What is the predicted direction of bias due to confounding?			
**2**.	**Bias in selection of participants into the study**			
2.1	Was selection of participants into the study (or into the analysis) based on variables measured after the start of the exposure?			
	**If N/PN to 2**.**1**: **go to 2**.**4**			
2.2	**If Y/PY to 2**.**1**: Were the post-exposure variables that influenced selection associated with exposure?			
2.3	**If Y/PY to 2**.**2**: Were the post-exposure variables that influenced eligibility selection influenced by the outcome or a cause of the outcome?			
2.4	Do start of follow-up and start of intervention coincide for most participants?			
2.5	**If Y/PY to 2**.**2 and 2**.**3**, **or N/PN to 2**.**4**: Were adjustment techniques used that are likely to correct for the presence of selection biases?			
	**Risk of bias judgement**			
	Optional: What is the predicted direction of bias due to selection of participants into the study?			
**3**.	**Bias in classification of exposures**			
3.1	Is exposure status well defined?			
3.2	Did entry into the study begin with start of the exposure?			
3.3	Was information used to define exposure status recorded prior to outcome assessment?			
3.4	Could classification of exposure status have been affected by knowledge of the outcome or risk of the outcome?			
3.5	Were exposure assessment methods robust (including methods used to input data)?			
	**Risk of bias judgement**			
	Optional: What is the predicted direction of bias due to measurement of outcomes or exposures?			
**4**.	**Bias due to departures from intended exposures**			
4.1	Is there concern that changes in exposure status occurred among participants?			
	**If your aim for this study is to assess the effect of initiating and adhering to an exposure** (**as in a per-protocol analysis**), **answer questions 4**.**2 and 4**.**3**, **otherwise continue to 4**.**4 if Y or PY to 4**.**1**.			
4.2	Did many participants switch to other exposures?			
4.3	Were the critical co-exposures balanced across exposure groups?			
4.4	**If Y/PY to 4**.**1**, **or Y/PY to 4**.**2**, **or 4**.**3**: Were adjustment techniques used that are likely to correct for these issues?			
	**Risk of bias judgement**			
	Optional: What is the predicted direction of bias due to departures from the intended exposures?			
**5**.	**Bias due to missing data**			
5.1	Were there missing outcome data?			
5.2	Were participants excluded due to missing data on exposure status?			
5.3	Were participants excluded due to missing data on other variables needed for the analysis?			
5.4	**If Y/PY to 5**.**1**, **5**.**2 or 5**.**3**: Are the proportion of participants and reasons for missing data similar across exposures?			
5.5	**If Y/PY to 5**.**1**, **5**.**2 or 5**.**3**: Were appropriate statistical methods used to account for missing data?			
	**Risk of bias judgement**			
	Optional: What is the predicted direction of bias due to missing data?			
**6**.	**Bias in measurement of outcomes**			
6.1	Could the outcome measure have been influenced by knowledge of the exposure received?			
6.2	Was the outcome measure sensitive?			
6.3	Were outcome assessors unaware of the exposure received by study participants?			
6.4	Were the methods of outcome assessment comparable across exposure groups?			
6.5	Were any systematic errors in measurement of the outcome unrelated to exposure received?			
	**Risk of bias judgement**			
	Optional: What is the predicted direction of bias due to measurement of outcomes?			
**7**	**Bias in selection of the reported result**			
	Is the reported effect estimate likely to be selected, on the basis of the results, from…?:			
7.1	…multiple outcome *measurements* within the outcome domain?			
**7.2**	…multiple *analyses* of the exposure-outcome relationship?			
7.3	…different *subgroups*?			
	**Risk of bias judgement**			
	Optional: What is the predicted direction of bias due to selection of the reported result?			
	**Overall risk of bias**			
	**Risk of bias judgement**			
	Optional: What is the overall predicted direction of bias for this outcome?			

As a next step, each pair of reviewers will independently re-assess the same set of NRSE following formal training and using a customized guidance sheet following the initial “without guidance” ROB assessments. At the end of the assessments, again the reviewers will meet to resolve conflicts and arrive at a consensus. All studies are assessed first without guidance, before any with-guidance assessments, to prevent the possibility of with-guidance assessment influencing without-guidance assessment. The principal investigator (MJ) at the coordinating center will coordinate this process among reviewers in the different participating centers.

Upon completion, the collaborating center will collect, organize, and transfer the ROB assessment data from various reviewers to an Excel workbook, prior to proceeding with the data analysis. We will then assess and report the IRR and ICR for ROB assessments “without guidance” and “with guidance,” separately.

#### Data analysis

An experienced biostatistician (RR) from the collaborating center will conduct all the analyses in collaboration with the other members of the research team. We will transfer all collected data from the Microsoft Excel workbook (Excel v14, Microsoft Corp., Redmond, WA, USA) to SAS (9.4), (SAS Institute Inc., Cary, NC, USA) for analysis. The kappa (*κ*) statistic is typically used to assess IRR as it corrects for the “chance” agreement between the two reviewers and allows for different types of disagreements to have differing weights [26]. The chance-agreement probability evaluated by the κ statistic assumes that all observed ratings may yield agreements by chance, thus leading to unpredictable results in the presence of high agreement between reviewers [27]. The AC_1_ statistic developed by Gwet [27] calculates the true overall chance agreement in the presence of high agreement reviewers, thus yielding values closer to “true” IRR [28]. We will also analyze the inter-consensus reliability (ICR) using Gwet’s AC_1_ statistic [27].

The agreements among reviewers (IRR and ICR) will be categorized as follows [29]: poor (0), slight (0.1-0.2), fair (0.21-0.4), moderate (0.41-0.6), substantial (0.61-0.8), or near perfect (0.81-0.99). We will tabulate the AC_1_ values and the 95% confidence intervals (CIs) separately (without or with guidance), as shown in Table 2. Additionally, we will assess the correlations between adjudications made during both the stages (“with guidance” and “without guidance”) for each of the reviewer to ensure that the effect of training and guidance is not biased.

**Table 2:** Reporting of IRR and ICR for ROB-NRSE (with or without guidance)

						IRR		ICR	
						Without customized guidance	With customized guidance	Without customized guidance	With customized guidance
Bias Domains	ROB Assessments					AC_1_ (95% CI)	AC_1_ (95% CI)	AC_1_ (95% CI)	AC_1_ (95% CI)
	L	M	S	C	NI				
Confounding									
Selection of participants									
Classification of exposures									
Departures from intended exposures									
Missing data									
Measurement of outcomes									
Selection of reported results									
Overall									

*L* low, *M* moderate, *S* serious, *C* critical, *NI* no information

### II: Concurrent validity

The second objective of this study is to evaluate the concurrent validity of the ROB instrument for NRS of exposures compared with NOS. Concurrent validity refers to how well a newly developed tool is correlated to similar domains of a widely used tool at the same point in time [30]. In other words, concurrent validity evaluates the extent to which there is concordance in judgment for similar domains in both the tools that are being compared [30]. Currently, there is no “gold standard” tool to asses ROB in NRSE. Hence, to assess the concurrent validity of the ROB instrument in NRS of exposures tool, we propose to use NOS, as it is the most commonly used quality assessment tool for NRSE that had been previously recommended by Cochrane [31].

In this cross-sectional study, we will explore the concordance between assessments made on similar domains in ROB-NRSE and NOS, and the overall assessments for each included NRSE.

#### Data collection

As mentioned previously, we will use a sample of NRSE (*n* = 44) for assessments of concurrent validity. We have compared and matched both NOS and the ROB instrument in NRS of exposures tool (as shown in Tables 3 and 4) to identify the items that *completely overlap*, *partially overlap*, or *unique* to each tool. Since the theoretical construct differs between NOS (methodological quality) and ROB-NRSE (ROB), we did not expect a complete match between all domains.

**Table 3:** Comparison of domains between NOS and ROB-NRSE

NOS		ROB-NRSE		Degree of overlap
Comparability	***C****:****Comparability of cohorts on the basis of the design or analysis***	Bias due to confounding	1.1: Is there potential for confounding of the effect of exposure in this study?	Unique
	*1a: Study controls for the most important factor*		1.2: Was the analysis based on splitting follow up time according to exposure received?	**Unique**
	*1b: Study controls for additional factor*		1.3. Were exposure discontinuations or switches likely to be related to factors that are prognostic for the outcome?	**Unique**
		*Baseline confounding only*	***1****.****4****:****Did the authors use an appropriate analysis method that controlled for all the important confounding domains****?*	**Complete overlap**
			1.5: Were confounding areas that were adjusted for measured validly and reliably by the variables available in this study?	**Unique**
			1.6. Did the authors avoid adjusting for post-exposure variables?	**Unique**
		*Time-varying confounding only*	1.7: Did the authors use an appropriate analysis method that adjusted for all the critically important confounding areas and for time-varying confounding?	**Unique**
			1.8: Were confounding areas that were adjusted for measured validly and reliably by the variables available in this study?	**Unique**
**Selection**	**S1**: **Representativeness of exposed cohort**	**Bias in selection of participants into the study**	2.1: Was selection of participants into the study (or into the analysis) based on variables measured after the start of the exposure?	**Unique**
	*1a*: *Truly representative*		2.2: Were the post-exposure variables that influenced selection associated with exposure?	**Unique**
	*1b*: *Somewhat representative*		2.3: Were the post-exposure variables that influenced eligibility selection influenced by the outcome or a cause of the outcome?	**Unique**
	*1c*: *Selected group of users*		2.4: Do start of follow-up and start of intervention coincide for most participants?	**Unique**
	*1d*: *No description of the derivation of the cohort*		2.5: Were adjustment techniques used that are likely to correct for the presence of selection biases?	**Unique**
	**S2**: **Selection of non-exposed cohort**			
	*2a*: *Drawn from the same community as the exposed cohort*			
	*2b*: *Drawn from a different source*			
	*2c*: *No description of the derivation of the non-exposed cohort*			
**Selection**	**S3**: **Ascertainment of exposure**	**Bias in classification of Exposures**	3.1: Is exposure status well defined?	**Unique**
	*3a*: *Secure record*		3.2: Did entry into the study begin with start of the exposure?	**Unique**
	*3b*: *Structured interview*		***3****.****3****:****Was information used to define exposure status recorded prior to outcome assessment****?*	**Partial overlap**
	*3c*: *Written self-report*		***3****.****4****:****Could classification of exposure status have been affected by knowledge of the outcome or risk of the outcome****?*	**Partial overlap**
	*3d*: *No description*		3.5: Were exposure assessment methods robust (including methods used to input data)?	
	***S4***: ***Demonstration of outcome of interest was not present at start of the study***			
	*4a*: *Yes*			
	*4b*: *No*			
		**Bias due to departures from intended exposures**	4.1: Is there concern that changes in exposure status occurred among participants?	**Unique**
			4.2: Did many participants switch to other exposures?	**Unique**
			4.3: Were the critical co-exposures balanced across exposure groups?	**Unique**
			4.4: Were adjustment techniques used that are likely to correct for these issues?	**Unique**
**Outcomes**	**O1**: **Was follow-up long enough for outcomes to occur** (**Yes/No**)	**Bias due to missing data**	***5****.****1****:****Were there missing outcome data****?*	**Partial overlap**
	***O3***: ***Adequacy of follow-up of cohorts***		5.2: Were participants excluded due to missing data on exposure status?	**Unique**
	*3a*: *Complete follow-up—all subjects accounted for*		5.3: Were participants excluded due to missing data on other variables needed for the analysis?	**Unique**
	*3b*: *Subjects lost to follow up unlikely to introduce bias -small number lost*		***5****.****4****:****Are the proportion of participants and reasons for missing data similar across exposures****?*	**Partial overlap**
	*3c*: *Follow up rate large* (*%*) *and no description of those lost*		5.5: Were appropriate statistical methods used to account for missing data?	**Unique**
	*3d*: *No statement*			
**Outcomes**	***O2***: ***Assessment of outcome***	**Bias in measurement of outcomes**	***6****.****1****:****Could the outcome measure have been influenced by knowledge of the exposure received****?*	**Partial overlap**
	*2a*: *Independent blind assessment*		6.2: Was the outcome measure sensitive?	**Unique**
	*2b*: *Record linkage*		***6****.****3****:****Were outcome assessors unaware of the exposure received by study participants****?*	**Partial overlap**
	*2c*: *Self report*		***6****.****4****:****Were the methods of outcome assessment comparable across exposure groups****?*	**Partial overlap**
	*2d*: *No description*		6.5: Were any systematic errors in measurement of the outcome unrelated to exposure received?	
		**Bias in selection of the reported result**	7.1: Is the reported effect estimate likely to be selected, on the basis of the results, from multiple outcome measurements within the outcome domain?	**Unique**
			7.2: Is the reported effect estimate likely to be selected, on the basis of the results, from multiple analyses of the exposure-outcome relationship?	**Unique**
			7.3: Is the reported effect estimate likely to be selected, on the basis of the results, from different subgroups?	**Unique**

**Table 4:** Similar items between NOS [7] and ROB-NRSE

	Similar Domains	ROB-NRSE (signaling questions)	NOS (domain items)	Degree of overlap
1.	**ROB-NRSE**: Bias due to confounding **NOS**: Comparability	1.4	C1a, C1b	Complete overlap
2.	**ROB-NRSE**: Bias in selection of participants **NOS**: Selection	-	-	Unique
3.	**ROB-NRSE**: Bias in classification of exposures **NOS**: Demonstration of outcome of interest was not present at start of the study	3.3, 3.4	S4a, S4b	Partial overlap
4.	**ROB-NRSE**: Bias due to deviations from intended exposures **NOS**: -	-	-	Unique
5.	**ROB-NRSE**: Bias due to missing data **NOS**: Adequacy of follow-up of cohorts	5.1, 5.4	O3a, O3b, O3c, O3d	Partial overlap
6.	**ROB-NRSE**: Bias in measurement of outcomes **NOS**: Assessment of outcome	6.1, 6.3	O2a, O2b, O2c, O2d	Partial overlap
7.	**ROB-NRSE**: Bias in selection of the reported result **NOS**: -	-	-	Unique

For the assessment of concurrent validity, one reviewer (MJ) with expertise in systematic reviews will assess NOS on a sample of NRSE (*n* = 44). We will then compare these NOS adjudications with the after-consensus adjudications of ROB-NRSE (done after customized training and guidance by two pairs of reviewers), for the same set of studies that were used for the ICR assessments.

We will calculate the correlation between the two tools for each of the domains and for the overall assessments. For comparison of overall assessments between the two tools, we will use the following algorithm: 0-2 stars in NOS will be considered similar to “critical ROB” in ROB-NRSE, 3-5 stars in NOS will be considered as similar to “serious ROB” in ROB-NRSE, 6-8 stars in NOS will be considered as similar to “moderate ROB” in ROB-NRSE, and 9 stars in NOS will be considered as similar to “low ROB” in ROB-NRSE. In addition, for any discordance observed between domains or overall assessment, we will explore the possible reasons and attempt to provide explanations.

#### Data analysis

An experienced biostatistician (RR) from the collaborating center will conduct all the analyses in collaboration with the other members of the research team. We will transfer all collected data from Excel workbook to SAS (9.4), (SAS Institute Inc., Cary, NC, USA) for analysis.

We will use the following algorithm for comparison between similar items (partially or completely overlapping) in the two tools (NOS and ROB-NRSE):

1. *For the* “*selection*” *domain in NOS*. Assessments with 4 stars will be considered equivalent to “low ROB” adjudication in ROB-NRSE. Assessments with 3 stars will be considered equivalent to “moderate ROB” adjudication in ROB-NRSE. Assessments with 2 stars will be considered equivalent to “serious ROB” adjudication in ROB-NRSE, and assessments with 0 or 1 star will be considered equivalent to “critical ROB” adjudication in ROB-NRSE.
2. *For the* “*comparability*” *domain in NOS*. Assessments with 2 stars will be considered equivalent to “low ROB” adjudication in ROB-NRSE. Assessments with 1 star will be considered equivalent to “moderate ROB” adjudication in ROB-NRSE. Assessments with 0 star will be considered equivalent to “serious or critical ROB” adjudication in ROB-NRSE.
3. *For the* “*outcome assessment*” *domain in NOS*. Assessments with 3 stars will be considered equivalent to “low ROB” adjudication in ROB-NRSE. Assessments with 2 stars will be considered equivalent to “moderate ROB” adjudication in ROB-NRSE. Assessments with 1 star will be considered equivalent to “serious ROB” adjudication in ROB-NRSE, and assessments with 0 star will be considered equivalent to “critical ROB” adjudication in ROB-NRSE.
4. The NOS domains with “no description/no statement” assessments will be considered equivalent to the “no information” adjudication in ROB-NRSE.

For measuring concordance or discordance between various domains of NOS and ROB-NRSE (i.e., to assess concurrent validity of ROB-NRSE ), we will use “Kendall’s tau,” a rank correlation coefficient statistic [32], and its 95% confidence intervals (for ordinal variables) for each domain and for the overall assessments.

### III: Evaluator burden

The time taken to apply any newly developed instrument is an important factor to consider, as it may contribute to significant burden on the evaluator/reviewer. It is also important to assess factors that could reduce the application time. In this study, we will compare the time taken to apply ROB-NRSE (without and with guidance), time taken by the reviewer pairs to arrive at a consensus (without and with guidance), and the time taken to apply NOS for comparison with ROB-NRSE.

#### Data collection process

Reviewers will record (using a digital clock) the time taken (in minutes) while applying (time to read article plus time to adjudicate) ROB-NRSE tool (without and with guidance), time taken for consensus, and the time taken to apply the NOS tool (time to read article plus time to adjudicate) for each included NRSE. The reviewers will use the Excel workbook created by the principal investigator to record the start time, end time, and total time to apply ROB-NRSE at the completion of the assessment for each NRSE and after the consensus process with the second reviewer. The reviewers will split the time to apply ROB-NRSE into the time taken to read the full text of the NRSE and the time taken for adjudications. The time to apply ROB-NRSE will begin when the reviewer begins reading the full texts of the NRSE and will end when decisions for all domains are completed and an overall ROB assessment for the study is established. The average overall time to apply ROB-NRSE for the same set of articles assessed by each reviewer will be calculated. In addition, we will also calculate the time taken to resolve conflicts and arrive at a consensus, and the overall time (time to apply plus time taken to arrive at a consensus) for each pair of reviewers. The time to arrive at a consensus will start when the two reviewers convene to resolve conflicts and will end when they arrive at a consensus.

#### Data analysis

An experienced biostatistician (RR) from the coordinating center will conduct all the analyses in collaboration with the other members of the research team. We will transfer all collected data from Excel workbook to SAS (9.4), (SAS Institute Inc., Cary, NC, USA) for analysis.

1. We will first summarize the average time (mean and SD) taken by the reviewers to assess ROB-NRSE without guidance and with guidance separately.
2. To analyze the impact of customized training and guidance on changes in evaluator burden (ROB-NRSE assessment time as well as the time taken by the reviewer pairs to arrive at consensus), we will compare two centers separately (*n* = 44 respectively). We will use generalized linear models to evaluate changes in time taken to assess ROB-NRSE after customized guidance (compared with without guidance). We will control for the correlation between reviewers using random effects. The distribution of outcome will be adjusted by using a link function.
3. To analyze the time taken to apply ROB-NRSE compared with NOS, we will use a fixed effect generalized linear model. The model distribution will be chosen by link function.

## Discussion

Systematic reviews including NRSE can provide valuable evidence on rare outcomes, adverse events, long-term outcomes, real-world practice, and in situations where RCTs are not available [9, 33]. It is very important to appraise the ROB in the included NRSE to have a complete understanding of the strengths and weaknesses of the overall evidence, as methodological flaws in the design or conduct of the NRSE could lead to biased effect estimates [9]. The newly developed ROB-NRSE could be a very useful tool for researchers in assessing risk of bias in NRSE when undertaking systematic reviews of NRSE. As such, it is important to evaluate the usability, reliability, and concurrent validity of this tool to help identify potential barriers and facilitators in applying this tool in a real-world setting.

In this cross-sectional study protocol, we describe the methods we will use to assess the inter-rater reliability, inter-consensus reliability, and the concurrent validity of ROB-NRSE. Our proposed study, upon completion, will provide empirical evidence on the IRR, concurrent validity, and the evaluator burden of ROB-NRSE.

### Strengths and challenges

Across the world, researchers, with a range of expertise, conduct systematic reviews that include NRSE. The ROB-NRSE tool was designed to be used by systematic reviewers with varied academic backgrounds and experience across multiple knowledge synthesis centers. A major strength of our study is that we will involve reviewers from multiple research teams with a range of expertise and academic backgrounds (highest degree attained) to apply and test ROB-NRSE, in order to simulate the real-world settings. We will also use a sample of NRSE that were not evaluated previously by the reviewers, in order to mimic what is typically encountered in a real-world setting. In addition, similar to what will be encountered in the real-world setting, we anticipate that the time taken to assess ROB might be longer for NRSE appraised at the beginning compared with those appraised later, due to increasing familiarity and a learning curve. To the best of our knowledge, there are no studies that have assessed the IRR, the ICR, the evaluator burden, the impact of additional training and guidance on IRR, ICR and the evaluator burden, and the construct validity of ROB-NRSE (comparison of ROB-NRSE with NOS). As with any elaborate tool, it is important to evaluate concerns regarding the practical use of ROB-NRSE. The findings of our cross-sectional study have a potential to elucidate the impact of training and development of customized guidance with decision rules on the IRR, ICR, and the evaluator burden of ROB-NRSE. Also, for data analysis, we will use the AC_1_ statistic developed by Gwet [27] to calculate true chance agreement in the presence of high agreement between reviewers, thus yielding values closer to “true” IRR for ROB-NRSE.

We anticipate the following limitations. For feasibility, the reviewers will only appraise ROB for a single outcome for each NRSE. This may be a limitation as reviewers in real-world settings that may need to appraise multiple outcomes for each of the included NRSE and the evaluator burden might differ slightly from the findings of this study. In a real-world setting, the training and customized guidance decision rules developed by the researchers for their own systematic reviews may differ from the one developed by the principal investigator of this study, and this may pose a challenge in the generalization of the findings of this study. For feasibility, we have proposed to use the same reviewers for both stages (without and with guidance), and we anticipate that this may bias the effect of training and guidance. However, we will address this limitation by assessing the correlations between adjudications made during the two stages, for each of the reviewers. A poor correlation between adjudications made during the two stages for a reviewer would indicate that the training and guidance have been useful.

As with any new tool, it is critical to assess the IRR, ICR, concurrent validity, and evaluator burden of ROB-NRSE, in order to improve the consistency of its application and its interpretation across various systematic reviews that include NRSE. We hope that the findings of this study will contribute to an improved understanding and better application of the ROB instrument for NRS of exposures tool.

## Knowledge Dissemination strategy

Systematic reviews serve as a source of knowledge and evidence to aid in the decision-making process. Our cross-sectional study addresses issues that may contribute to the quality of the evidence synthesized by the systematic review and thus will be of great interest to all stakeholders such as clinicians, decision-makers, patients, and the general-public through GRADE assessments of the quality of the evidence. It will also be of great interest to researchers conducting Cochrane and non-Cochrane systematic reviews to improve their understanding regarding the practical use of the ROB-NRSE tool. We plan to disseminate the results of our cross-sectional study by presenting the study results at various conferences (e.g., Cochrane Colloquium) by publishing study results in academic journals and by spreading the message through social media (e.g., Twitter).

## Data Availability

Not applicable.

## Abbreviations

GRADE: Grading of Recommendations, Assessment, Development and Evaluation
ICR: Inter-consensus reliability
IRR: Inter-rater reliability
NLM: National Library of Medicine
NOS: Newcastle-Ottawa Scale
NRS: Non-randomized studies
NRSE: Non-randomized studies of exposures
NRSI: Non-randomized studies of interventions
RCT: Randomized controlled trials
ROB: Risk of bias
ROBINS-E: Risk of Bias in Non-randomized Studies of Exposures
ROBINS-I: Risk of Bias in Non-randomized Studies of Interventions
SD: Standard deviation
UK: United Kingdom
USA: United States of America
*κ*: Kappa statistic
